# Effect of Adding Telephone-Based Brief Coaching to an mHealth App (*Stay Strong*) for Promoting Physical Activity Among Veterans: Randomized Controlled Trial

**DOI:** 10.2196/19216

**Published:** 2020-08-04

**Authors:** Laura J Damschroder, Lorraine R Buis, Felicia A McCant, Hyungjin Myra Kim, Richard Evans, Eugene Z Oddone, Lori A Bastian, Gwendolyn Hooks, Reema Kadri, Courtney White-Clark, Caroline R Richardson, Jennifer M Gierisch

**Affiliations:** 1 Veterans Affairs Center for Clinical Management Research Ann Arbor Healthcare System Ann Arbor, MI United States; 2 University of Michigan Department of Family Medicine Ann Arbor, MI United States; 3 Veterans Affairs Durham Center of Innovation to Accelerate Discovery and Practice Transformation Durham Veterans Affairs Health Care System Durham, NC United States; 4 Division of General Internal Medicine Department of Medicine Duke University Medical Center Durham, NC United States; 5 Veterans Affairs Pain Research, Informatics, Multimorbidities, and Education Center Veterans Affairs Connecticut West Haven, CT United States; 6 Division of General Internal Medicine Department of Medicine Yale University West Haven, CT United States; 7 Department of Population Health Sciences Duke University Medical Center Durham, NC United States

**Keywords:** exercise, veterans, smartphones, wearable physical activity tracker, behavior change, mobile phone, online, app, mobile app, wearable

## Abstract

**Background:**

Though maintaining physical conditioning and a healthy weight are requirements of active military duty, many US veterans lose conditioning and rapidly gain weight after discharge from active duty service. Mobile health (mHealth) interventions using wearable devices are appealing to users and can be effective especially with personalized coaching support. We developed *Stay Strong*, a mobile app tailored to US veterans, to promote physical activity using a wrist-worn physical activity tracker, a Bluetooth-enabled scale, and an app-based dashboard. We tested whether adding personalized coaching components (*Stay Strong+Coaching*) would improve physical activity compared to *Stay Strong* alone.

**Objective:**

The goal of this study is to compare 12-month outcomes from *Stay Strong* alone versus *Stay Strong+Coaching.*

**Methods:**

Participants (n=357) were recruited from a national random sample of US veterans of recent wars and randomly assigned to the *Stay Strong* app alone (n=179) or *Stay Strong+Coaching* (n=178); both programs lasted 12 months. Personalized coaching components for *Stay Strong+Coaching* comprised of automated in-app motivational messages (3 per week), telephone-based human health coaching (up to 3 calls), and personalized weekly goal setting. All aspects of the enrollment process and program delivery were accomplished virtually for both groups, except for the telephone-based coaching. The primary outcome was change in physical activity at 12 months postbaseline, measured by average weekly Active Minutes, captured by the Fitbit Charge 2 device. Secondary outcomes included changes in step counts, weight, and patient activation.

**Results:**

The average age of participants was 39.8 (SD 8.7) years, and 25.2% (90/357) were female. Active Minutes decreased from baseline to 12 months for both groups (*P*<.001) with no between-group differences at 6 months (*P*=.82) or 12 months (*P*=.98). However, at 12 months, many participants in both groups did not record Active Minutes, leading to missing data in 67.0% (120/179) for *Stay Strong* and 61.8% (110/178) for *Stay Strong+Coaching*. Average baseline weight for participants in *Stay Stron*g and *Stay Strong+Coaching* was 214 lbs and 198 lbs, respectively, with no difference at baseline (*P*=.54) or at 6 months (*P*=.28) or 12 months (*P*=.18) postbaseline based on administrative weights, which had lower rates of missing data. Changes in the number of steps recorded and patient activation also did not differ by arm.

**Conclusions:**

Adding personalized health coaching comprised of in-app automated messages, up to 3 coaching calls, plus automated weekly personalized goals, did not improve levels of physical activity compared to using a smartphone app alone. Physical activity in both groups decreased over time. Sustaining long-term adherence and engagement in this mHealth intervention proved difficult; approximately two-thirds of the trial’s 357 participants failed to sync their Fitbit device at 12 months and, thus, were lost to follow-up.

**Trial Registration:**

ClinicalTrials.gov NCT02360293; https://clinicaltrials.gov/ct2/show/NCT02360293

**International Registered Report Identifier (IRRID):**

RR2-10.2196/12526

## Introduction

### Background

Adequate levels of physical activity (PA) reduce the risk of many diseases including diabetes, obesity, cardiovascular disease, dementia, and many cancers. Furthermore, adequate levels contribute to significant improvements in quality of life by improving sleep and physical function, preventing falls, and improving pain management. Inadequate PA is one of the top drivers of premature death. Despite strong evidence for PA’s beneficial effects, most adult men (74%) and women (81%) in the United States do not meet national recommendations for PA levels [1] (150 minutes of moderate activity or 90 minutes of vigorous activity per week [2]).

A lack of PA is especially prevalent among veterans of the US Armed Forces. The type and intensity of physical activities that veterans engage in too often abruptly reduces as they transition from active duty to postdeployment civilian life. The relatively unstructured nature of postdeployment life and illnesses or injuries sustained during military service may contribute to this shift in activity levels [3]. Younger veterans involved in the Afghanistan and Iraq wars may also have work-life balance issues related to child and older adult care issues, and challenges integrating into civilian life because of high physical and mental health burdens (eg, chronic pain, mental illness, substance abuse) [4,5]. Additionally, in one large cohort, 65.8% of men and 46.7% of women were overweight or obese at their first postdeployment visit in the Veteran Health Administration, an additional barrier to engaging in PA [6].

One potential strategy for increasing PA among veterans is using readily available consumer-grade wearable PA sensors and monitoring devices. A solid foundation of evidence demonstrates shorter-term effectiveness of internet-mediated interventions [7-17], particularly when combined with wearable PA sensors, tailored motivational messaging, and coaching [18-20]. By leveraging the broad availability of mobile sensors, we can increase access to interventions aimed at increasing PA levels [21-23]. The evidence-base for mobile health (mHealth) interventions, however, is largely based on small trials with short-term follow-up [13,21]; longer-term engagement with mHealth programs is not always sustained [24-26]. Human-based or automated health coaching, which has produced positive lifestyle changes across a wide range of populations [26-29], has potential for sustaining longer-term adherence, engagement, and outcomes.

Several studies using mobile apps to promote weight loss and increase PA have reported high rates of attrition, with participants dropping out after the first month [11]. Without active adherence and engagement by participants, the mHealth intervention is unlikely to be effective [30]. A potential strategy to increase adherence and engagement with, and effectiveness of mHealth interventions, is to add health coaching, including telephone-based lifestyle coaching delivered by humans [31-35]. Health coaching is a patient-centered, collaborative model grounded in theories of health behavior change where coaches work in partnership with patients using motivational interviewing, goal setting, and problem solving as key strategies. Across a wide variety of populations, health coaching has produced positive impacts on lifestyle modifications [27-29]. In a recent trial, a relatively low-intensity dose (2 coaching calls at 1 and 4 weeks postbaseline) of telephone-based coaching in conjunction with use of an online risk assessment tool resulted in increased engagement in lifestyle change programs and increased patient activation in a trial among US veteran participants, compared to use of the online risk assessment alone [27-29]. Thus, adding coaching features such as telephone-based human coaching, extended with personalized automated messaging to help address barriers and provide motivation and personalized weekly goals, may further enhance the impact of mHealth interventions.

### Study Objective

The objective of this study is to determine whether PA levels would improve at 12 months with a wearable activity tracker combined with health coaching versus a wearable activity tracker alone, among US veterans of recent Afghanistan or Iraq wars. PA levels were measured by Active Minutes, as recorded by the Fitbit Charge 2, a consumer-grade mobile PA sensor that was widely available at the time of this study.

## Methods

### Study Design

This is a comparative effectiveness randomized controlled trial comparing two 12-month programs: *Stay Strong*, an mHealth intervention using a wearable activity tracker with a dashboard available through a smartphone app along with a Bluetooth-enabled weight scale, versus *Stay Strong+Coaching,* comprising *Stay Strong* plus human health coaching provided over the telephone and in-app automated weekly personalized PA goals, motivational messages, and personalized weekly PA goals. A summary of the methods is provided here; our published protocol provides more details [36].

### Recruitment

A stratified random sample of administrative medical record data for US veterans of recent wars including Operation Enduring Freedom (OEF), Operation Iraqi Freedom (OIF), and Operation New Dawn (OND) residing within the United States was used to identify potentially eligible individuals. We oversampled women to ensure they comprised at least 20% of participants. Inclusion criteria were online confirmation of OEF, OIF, or OND veteran status; identifying a Veterans Health Administration (VHA) health care provider as being responsible for their medical care; having interest in starting a PA program within the next 30 days; having access to a computer with internet connection and a working USB port; having a smartphone with a compatible iOS or Android operating system; and being younger than 65 years (because the interventions were targeted to OEF, OIF, and OND veterans who would typically be younger than 65 years). Individuals were excluded if they reported that a health care provider had told them that it was currently unsafe to exercise in an unsupervised or unmonitored setting, had a history of eating disorders or a BMI<20, were not competent to consent for themselves to a research study, or wore a PA sensor within the last 30 days.

Invitation letters briefly describing the study included a URL with an individualized code to access an online portal. This was the first study within the Veterans Affairs (VA) that relied completely on online technology-mediated approaches for recruitment, consent, Health Insurance Portability Accountability Act (HIPAA) authorization, enrollment, delivery of the interventions, and conduct of the program (see [37] for content). Telephonic support was available to participants as needed for technical support.

After consent, participants completed a baseline survey. At the end of the survey, online instructions asked participants to download and install the *Stay Strong* app on their smartphone via the Google Play (Android smartphone users) or Apple (iPhone users) stores. When *Stay Strong* was successfully installed, a package was shipped containing instructions, their Fitbit Charge 2 device, a Bluetooth-enabled weight scale, and a USB dongle for syncing their Fitbit device using a USB-enabled computer. Individuals were instructed to sync their Fitbit device via the Fitbit Connect software using a Bluetooth-enabled dongle that was plugged into a USB port on a computer. This configuration was necessary to comply with VHA data security and confidentiality standards. Syncing was typically completed within minutes of initiation. Individuals were also instructed to configure their Bluetooth-enabled weight scale by pairing their smartphone with the scale. In-app instructions were provided to walk the user through step-by-step so that data received via Bluetooth connection would be recorded by the *Stay Strong* app and displayed by the in-app dashboard. Synching typically lasted seconds.

Participants authorized Vibrent, Inc (the developer of the *Stay Strong* server platform) [38] to sync and access their Fitbit data. Their Fitbit device had a “Do Not Remove” sticker, covering the device’s display during the baseline period before randomization.

When at least 5 valid days of data (days when at least 5 Lightly Active Minutes were recorded) within a 7-day period were synced to the study server, the individual was randomized in a 1:1 ratio, to *Stay Strong* or *Stay Strong+Coaching*. All the study staff were blinded to the randomization list that was generated by the study biostatistician. After the participant was assigned to an arm, their smartphone-based mobile app was updated to reflect their assigned program (*Stay Strong* or *Stay Strong+Coaching*) and they were instructed to remove the sticker that covered their Fitbit display.

Follow-up surveys were administered at 6- and 12-months postbaseline. Respondents were mailed a US $25 Amazon gift card for each completed follow-up survey. All participants kept their Fitbit device and scale after their program ended.

### Institutional Review Board Approval and Ethical Considerations

Ethical oversight was provided by VHA’s Central Institutional Review Board that approved the protocol. A copy of the approved study protocol is available online [37]. Participants were randomized between October 11, 2017, and May 31, 2018. The last participants finished their program on July 9, 2019.

### Interventions

Table 1 lists program components for the two trial arms. The *Stay Strong* programs both lasted 12 months. Designs were informed by experiences of veterans in a prior study [39] and by the self-regulation theory and the information-motivation-behavioral skills model [40-43], which describe processes of behavior change mediated through goal attainment and skills mastery, and acknowledges the central role of self-efficacy in sustaining change in PA [41,44-47]. Our published protocol provides more details about behavior change techniques incorporated into the programs [36]. Detailed functional requirements, screenshots, along with the full library of messages sent to participants are available online [37]. The Fitbit Charge 2 provided detailed minute-by-minute self-monitoring information through objective measurement of PA. A veterans’ work group provided input into the logistics of intervention delivery and enrollment processes. Feedback on an early version of *Stay Strong+Coaching* was elicited from a convenience group of testers employed at the VA and veterans who served on an advisory panel for this grant, several of whom were OEF, OIF, or OND veterans. No changes were made to *Stay Strong* during the course of this trial.

**Table 1:** *Stay Strong* components by trial arm.

Component	*SS^a^* arm	*SS+Coaching* arm	Intensity	Duration	Mode
Objective physical activity monitoring (Fitbit Charge 2 and data visualizations within *SS*)	✓	✓	Fitbit worn daily, and data syncing at least 1/week	1 year	Fitbit worn on wrist; data visualizations available within *SS* app
Weight self-monitoring (scale and weight data visualizations within *SS*)	✓	✓	Weight measured weekly with data syncing at least 1/week	1 year	Data visualizations available within *SS* app
Administrative message reminders (reminders for Fitbit and weight scale syncing, adverse event reporting, and data assessments)	✓	✓	One message less than 230 characters	As needed over 1 year	Push notification on phone
Automated personalized goal setting		✓	Weekly, based on previous weeks’ physical activity data	1 year	Abbreviated phone push notification and message with image within *SS* app
Automated messages: nonpersonalized		✓	1 message up to 225 characters, 3/week	1 year	Abbreviated phone push notification and full message with visual image within SS app
Automated messages: personalized based on self-reported barriers		✓	1 message up to 225 characters, 3/week	1 year	In-app and smartphone notification with image
Telephone-based lifestyle coaching		✓	Up to 30 min	2 calls plus an optional 3rd call in the first 9 weeks	Telephone

^a^*SS*: *Stay Strong*.

#### *Stay Strong* Intervention

The *StayStrong* program lasted 12 months and comprised of a Fitbit device to capture PA, a Bluetooth-enabled weight scale, and a smartphone app with a dashboard showing key metrics over time (Active Minutes, miles, steps, stairs, and heart rate zone). The Fitbit Charge 2 device is a wrist-worn PA monitoring device that continuously logs PA. Participants were encouraged to wear the Fitbit device during waking hours for the study duration and to upload device data at least weekly via the Fitbit Connect software and USB port on their computer. The smartphone app displayed PA data in 1- to 4-week increments (or most recent valid week).

Data from the Bluetooth-enabled weight scale (A&D Deluxe Connected Weight Scale UC-352BLE) was synced with *Stay Strong* or could be manually entered. The smartphone app displayed weight data in 1- to 4-week increments. Participants were asked to weigh themselves and sync their scale at least weekly during the duration of their program.

During the study period, all participants received automated administrative messages including reminders to report adverse events every 90 days and reminders to complete 6- and 12-month survey assessments.

#### Stay Strong+Coaching Intervention

In addition to the *Stay Strong* components, participants received personalized coaching, which comprised of automated in-app motivational messages (3/week for the duration of the 12-month program), telephone-based human health coaching (up to 3 calls, spaced over the first 9 weeks), and personalized weekly goal setting. The coaching telephone calls were designed to motivate participants by helping them develop goals and action plans to achieve Fitbit-derived PA goals, problem solve barriers to achieving PA goals, and understand features of the *Stay Strong* app, with an emphasis on interpreting Fitbit PA data shown in their dashboard to monitor their progress. PA goals were computed by increasing each new daily PA goal by 5 Active Minutes based on previous week’s (or most recent) synced Active Minutes, not to exceed 60 Active Minutes per day. Participants received three messages per week delivered within the app and via push notification to the smartphone; most were nonpersonalized, but a subset were personalized to address specific barriers identified by each participant. To maintain interest and engagement in the messages throughout the 12 months, we randomly timed in-app message delivery during the day Monday through Saturday. Messages comprised of a maximum 225 characters and were designed to help participants stay engaged and learn more about topics including: exercise, healthy eating, initiating behavior change, pain, inspirational quotes, maintaining behavior change, weight loss and management, heart rate monitoring, and appropriate athletic gear. Additionally, at baseline and 6 months, participants were asked to choose up to four barriers that most prevented increasing PA from a list of 11 prespecified barriers (lack of time, social influence, lack of energy, lack of willpower or motivation, fear of injury or pain, lack of resources, family obligations, weather conditions, depression, accountability or external motivation, and disability). The prespecified list was developed based on work by Sallis and colleagues [48,49] and highlighted by the Centers for Disease Control and Prevention, plus the addition of disability.

### Outcomes

The primary outcome was Active Minutes per week, as recorded by the Fitbit Charge 2 device, for 12 months following randomization. To report PA levels (eg, Active Minutes, steps), participants synced their Fitbit device as often as desired. We encouraged participants to sync at least once per week. Active Minutes is a proprietary measure that captures the number of minutes of continuous moderate-to-vigorous exercise when sustained for at least 10 minutes [50]. Secondary outcomes included step counts (reported through syncing the Fitbit device), weight loss, and patient activation. Weight was to be recorded by the Bluetooth-enabled scale; participants were encouraged to record their weight at least weekly. However, most participants did not sync their scales and, thus, did not provide weights. Therefore, for the comparison of weight change rates, we conducted an alternative analysis with weights captured by the VA administrative medical record data, and baseline weights were self-reported at the time of enrollment. Patient activation was assessed by online questionnaire at baseline and at 6 and 12 months postbaseline using the self-reported 13-item Patient Activation Measure (PAM) [51]. The PAM assesses an individual’s knowledge, skills, beliefs, and confidence for managing their own health. PAM scores have high construct validity and have been positively associated with engagement in healthier lifestyle behaviors [52].

### Sample Size

Our sample size calculations were based on unpublished data from a pilot study. A 10-minute differential improvement at 12 months was set as a minimal clinically important difference. We anticipated a baseline mean of 53 minutes, a standard deviation of 28 minutes in both treatment groups, and *r*=0.46 correlation between baseline and 12 months. Because of the lengthy enrollment process, we expected up to 50% dropout during the consent and preparation phases, and assumed 25% dropout after enrollment during the 12-month program. We aimed to randomize 350 patients (175 per group) to detect a 10-minute difference in improvement at 12 months based on a 5% significance level 2-sided test using analysis of covariance (ANCOVA) with 90% power.

### Statistical Analysis

Primary analyses were based on intent-to-treat focusing on the effect of *Stay Strong+Coaching* compared to *Stay Strong* alone on change in PA from baseline to 12 months postrandomization. Women who self-reported pregnancy at any of the three primary assessment times (baseline, 6-month, 12-month) were not included in the analyses for PA and weight; 1 participant indicated pregnancy at the 6-month follow-up. Summary statistics (eg, means, medians, and proportions) were used to describe all study variables including outcome measures for overall study participants, by study arm, and at each of the three primary assessment times. Adjusted between-arm difference in Active Minutes at both 6 and 12 months were compared and estimated based on a mixed model using data at the three primary assessment times. Between-arm comparison was also done using a mixed model with all longitudinally assessed weekly averages of Active Minutes as dependent variables. The model included time (weeks since randomization), treatment arm (*Stay Strong+Coaching* arm) indicator, an interaction of time by treatment arm as primary predictors, and random intercepts and slopes. The model was also adjusted for baseline Active Minute goal and stratification factors of sex and smartphone operating system. A test of significant slope of the interaction term was used to assess if the rate of change in Active Minutes over the study period differed between treatment arms. Secondary outcomes of steps and weights were analyzed similarly using data at primary assessment times, as well as full weekly data, and patient activation was analyzed using data at three primary assessment times.

Several alternative analyses were conducted to ensure consistency in our main results. Due to high skewness of the PA data, we modeled weekly Active Minutes after log-transformation and step counts after taking the square root. For PA data, we also used robust regression based on the median, minimizing the sum of the absolute deviation from the estimate of the center.

Additionally, to account for a substantial amount of missing follow-up data, primary analyses were repeated using weighted likelihood methods to give more weights to individuals who were more likely to miss 12-month outcomes. Weights were estimated from a penalized likelihood (least absolute shrinkage and selection operator) logistic regression model, with missing 12-month data as the response variable and with baseline sociodemographic characteristics as predictors of missing 12-month data. “Do Not Remove” stickers covered each participant’s Fitbit screen to prevent feedback that may motivate higher levels of PA even with instructions to maintain normal levels of activity. Alternative analyses were conducted to test for “reactivity,” where participants may have increased their activity levels despite these precautions. This “reactivity” often manifests as unusually high or low activity levels with use of a new device like the Fitbit Charge 2 used in this study. If this occurred, PA may decline to previously normal levels by the second week. To assess reactivity, we re-estimated the between-arm difference in Active Minutes at 12 months after replacing baseline data with the second week data. The a priori level for statistical significance was a 2-sided *P*<.05. For all analyses, R version 3.6.0 (R Foundation for Statistical Computing) was used. All code and detailed results are available online at [37].

## Results

### Participant Characteristics

**Figure 1:**
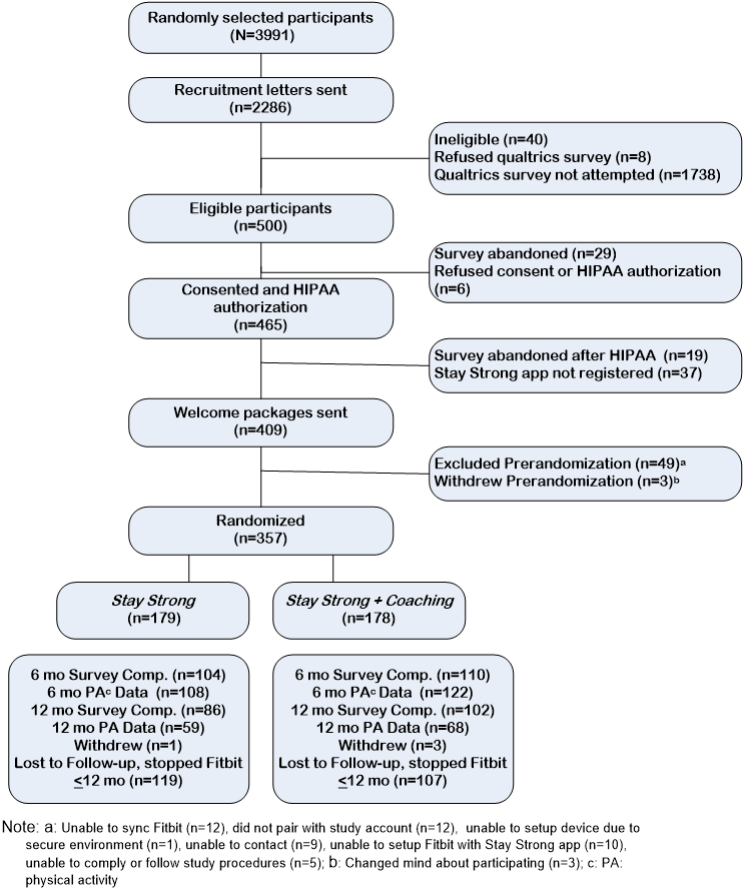
CONSORT flow of recruitment through randomization.

Letters with the VHA letterhead along with a logo specially designed for *Stay Strong* were mailed to 2286 randomly selected US veterans, of whom 17.9% (409) were eligible, completed consent, provided HIPAA authorization, and to whom welcome packages were sent containing their Bluetooth scale and Fitbit device along with instructions for use (see Figure 1). Of those 409 participants, 357 (87.3%) successfully set up their devices and synced a week of valid PA data and were randomized to *Stay Strong* (n=179) or *Stay Strong+Coaching* (n=178).

Table 2 shows baseline participant characteristics overall and by study arm. Of the 357 participants, the average age was 39.8 years, 90 (25.2%) were female, 231 (64.7%) were non-Hispanic White, 248 (69.5%) were married, 215 (60.2%) had children in the household, 156 (40.9%) had a bachelor’s or higher college degree, and 48 (13.4%) were current smokers. Additionally, based on administrative medical record data, 191 (53.5%) were diagnosed with posttraumatic stress disorder (PTSD), 118 (35%) had diagnoses of clinically significant depression, 155 (43%) reported moderate or severe pain, 95 (26.6%) reported clinically significant alcohol misuse based on Alcohol Use Disorders Identification Test Version C score, and 204 (57.1%) were obese (BMI≥30).

**Table 2:** Baseline characteristics of participants (N=357).

Characteristics			Overall (N=357)	*SS*^a^ (n=179)		*SS+Coaching* (n=178)
**Sociodemographic factors**						
	Sex (female), n (%)		90 (25.2)	43 (24.0)		47 (26.4)
	Age (years), mean (SD)		39.8 (8.7)	40.4 (9.0)		39.2 (8.4)
	**Race/ethnicity, n (%)**					
		Non-Hispanic White	231 (64.7)		114 (63.7)	117 (65.7)
		Non-Hispanic Black	47 (13.2)		27 (15.1)	20 (11.2)
		Other	79 (22.1)		38 (21.2)	41 (23.0)
	Married, n (%)		248 (69.5)	128 (71.5)		120 (67.4)
	Have children in household, n (%)		215 (60.4)	117 (65.7)		98 (55.1)
	**Education, n (%)**					
		High school graduate/equivalent (GED^b^) or less	23 (6.4)		7 (3.9)	16 (9.0)
		Some college, trade/vocational, associate’s degree	188 (52.7)		98 (54.7)	90 (50.6)
		Bachelor’s degree	94 (26.3)		47 (26.4)	47 (26.3)
		Postgraduate work or graduate degree	52 (14.6)		27 (15.1)	25 (14.0)
	Full-time employment, n (%)		202 (56.6)	97 (54.2)		105 (59.0)
	Inadequate income^c^, n (%)		49 (13.7)	27 (15.1)		22 (12.4)
**Health and comorbidities**						
	**General health^d^, n (%)**					
		Excellent	16 (4.5)		16 (4.5)	8 (4.5)
		Very good	74 (20.7)		74 (20.7)	36 (20.2)
		Good	150 (42.0)		150 (42.0)	71 (39.9)
		Fair	97 (27.2)		97 (27.2)	50 (28.1)
		Poor	20 (5.6)		20 (5.6)	13 (7.3)
	Moderate/severe pain^e^, n (%)		155 (43.4)	75 (41.9)		80 (44.9)
	Diabetes^f^, n (%)		27 (7.6)	16 (8.9)		11 (6.2)
	Hypertension^f^, n (%)		63 (17.6)	31 (17.3)		32 (18.0)
	Posttraumatic stress disorder diagnosis^f^, n (%)		191 (53.5)	97 (54.2)		94 (52.8)
	**Depression scale (PHQ-8^g^), median (IQR)**		6.5 (3.0-12.0)	6.0 (3.0-11.0)		7.0 (3.0-13.0)
		Clinically significant depression^h^, n (%)	118 (34.9)		57 (33.9)	61 (35.9)
**Lifestyle and related factors**						
	Patient Activation Measure, mean (SD)^i^		70.2 (11.9)	70.3 (12.2)		70.1 (11.7)
	AUDIT-C^j^≥4 (male); AUDIT-C≥3 (female), n (%)		95 (26.6)	42 (23.5)		53 (29.8)
	Current smoker^k^, n (%)		48 (13.4)	22 (12.3)		26 (14.6)
	Weight (lbs), mean (SD)		210.4 (44.9)	208.5 (39.5)		212.3 (49.8)
	**BMI (kg/m^2^), mean (SD)**		31.1 (5.7)	30.9 (5.3)		31.2 (6.2)
		<25	38 (10.6)		16 (8.9)	22 (12.4)
		25-29	115 (32.2)		57 (31.8)	58 (32.6)
		30-34	110 (30.8)		62 (34.6)	48 (27.0)
		35-39	70 (19.6)		34 (19.0)	36 (20.2)
		≥40	24 (6.7)		10 (5.6)	14 (7.9)
**Technology use**						
	Comfort using the internet^l^, mean (SD)		32.6 (3.8)	32.4 (4.0)		32.8 (3.6)
	Phone type: iPhone^m^, n (%)		174 (48.7)	89 (49.7)		85 (47.8)
	**Physical activity device use^n^, n (%)**		164 (45.9)	74 (41.3)		90 (50.6)
		Prior experience with Fitbit use, n (%)	95 (26.6)		44 (24.6)	51 (28.7)

^a^*SS*: *Stay Strong*.

^b^GED: General Education Development.

^c^Responded yes to “Must cut back on things to pay bills or have difficulty paying bills at the end of the month.”

^d^12-Item Short-Form Health Survey [53].

^e^Pain intensity ≥4 out of 11-point scale.

^f^See [37] for specific diagnosis codes used for defining this category.

^g^PHQ-8: Patient Health Questionnaire Depression Scale.

^h^PHQ-8≥10 [54].

^i^Patient Activation Measure-13 score [51].

^j^AUDIT-C: Alcohol Use Disorders Identification Test Version C.

^k^Combination of “Smoke every day” and “Smoke some days.”

^l^Score range from 7 to 35; a higher score corresponds to more comfort.

^m^In comparison to Android.

^n^Prior experience with a physical activity device (eg, Fitbit, Apple Watch).

At baseline, 103/179 (57.9%) and 96/178 (53.9%) of participants in *Stay Strong* and *Stay Strong+Coaching* recorded more than 150 minutes of weekly Active Minutes (*P*=.46). All participants recorded Active Minutes during their baseline week, but by 12 months postrandomization, most participants were not synching their Fitbit, thus PA data were not available; specifically, though 230/357 (64.4%) participants provided synced data at 6 months, only 127 (35.6%) did so at 12 months.

### Primary Outcomes

Adjusted mean Active Minutes, based on a repeated measures ANCOVA model, showed no between-arm differences at 6 months (*P*=.82) or 12 months (*P*=.98). Mean weekly Active Minutes reported by Fitbit devices declined in both arms. A mixed model based on weekly longitudinal Active Minutes data revealed Active Minutes decreased significantly over the 12 months in the *Stay Strong* group (weekly slope=–3.04, *P*<.001), with no significant difference in the rate of decrease in the two study arms (*P*=.40 for the interaction of time by the *Stay Strong+Coaching* arm indicator). Multiple alternate analytic models resulted in similar findings with neither clinically, nor statistically, significant differences in Active Minutes between study arms. For example, a mixed model weighted by the estimated probability of missing 12-month data showed significantly decreasing Active Minutes over time (*P*<.001), but no difference in the rate of decrease in Active Minutes between the two arms (*P*=.37). We also tested and adjusted for “reactivity,” given the high baseline levels of Active Minutes recorded to answer the question. We first assessed whether participants increased their normal PA levels in their baseline week despite masking feedback on their Fitbit device by covering their Fitbit display with a “Do Not Remove” sticker that prevented users from seeing and reacting to PA levels recorded by the Fitbit. If reactivity was present, then we would expect PA levels to decrease in the following weeks [55,56]. Our analyses revealed that Active Minutes decreased slightly from the first to the second week; however, analysis where second week data replaced the baseline data did not alter findings.

### Secondary Outcomes

We found no significant differences between arms in any secondary outcomes including step counts (*P*=.08), weight (*P*=.55), or patient activation *(P*=.98) at the 12-month follow-up (Table 3). The between-arm difference in the predicted mean at 12 months was 1009 steps per day, adjusting for sex, type of smartphone, and baseline goal. For step counts per day, averaged over a week, crude means declined from 8163 steps per day at baseline to 5736 at 12 months in the *Stay Strong* arm, and from 7571 to 5638 in the *Stay Strong+Coaching* arm. Multiple alternate models based on weekly step counts all showed significantly decreasing step counts over the 12 months in the *Stay Strong* group (*P*<.001), with no significant difference in the rate of decrease in the two study arms.

**Table 3:** Primary outcome of Active Minutes and secondary outcomes of steps, weight, and patient activation for each major assessment time.

Outcomes		*Stay Strong* (n=179), mean^a^ (95% CI)	*Stay Strong+Coaching* (n=178), mean^a^ (95% CI)	Between-group difference, mean^b^ (SE)	*P* value^c^
**Active Minutes per week**					
	Baseline (N=357)	255 (216-295)	240 (201-280)	8.63 (23.0)	.71
	6-month (n=230)	225 (175-276)	234 (186-281)	–10.0 (24.2)	.68
	12-month (n=127)	190 (121-258)	199 (136-263)	–29.4 (41.2)	.48
**Steps per week**					
	Baseline (N=357)	8163 (7531-8795)	7571 (6938-8205)	152 (465)	.74
	6-month (n=230)	6351 (5537-7165)	6563 (5797-7328)	–841 (551)	.13
	12-month (n=127)	5736 (4635-6837)	5638 (4612-6663)	–1009 (583)	.08
**Weight (lbs)**					
	Baseline (N=357)	214 (200-228)	198 (182-215)	–3.5 (4.8)	.46
	6-month (n=97)	217 (205-228)	206 (193-219)	–3.3 (4.9)	.49
	12-month (n=65)	221 (206-235)	217 (203-232)	–3.1 (5.3)	.55
**Patient activation**					
	Baseline (n=315)	70.4 (68.5-72.2)	70.1 (68.3-72.0)	0.20 (1.35)	.88
	6-month (n=198)	68.0 (65.7-70.4)	66.9 (64.5-69.3)	0.91 (1.65)	.58
	12-month (n=171)	69.4 (66.8-72.1)	69.2 (66.7-71.6)	–0.04 (1.77)	.98

^a^Crude means. n in the first column represent the number of participants with available data for crude means.

^b^Calculated as the estimated marginal mean difference (*Stay Strong* group – *Stay Strong+Coaching* group) based on a model fit using all available data (n=179 for *Stay Strong* and n=178 for *Stay Strong+Coaching*) and adjusting for baseline goal, sex, and operating system type for all outcomes except for patient activation, which relies on n’s listed in the first column for between-group difference and *P* values.

^c^For between-group difference, adjusted for comparing a family of 3 estimates.

### Adherence

**Figure 2:**
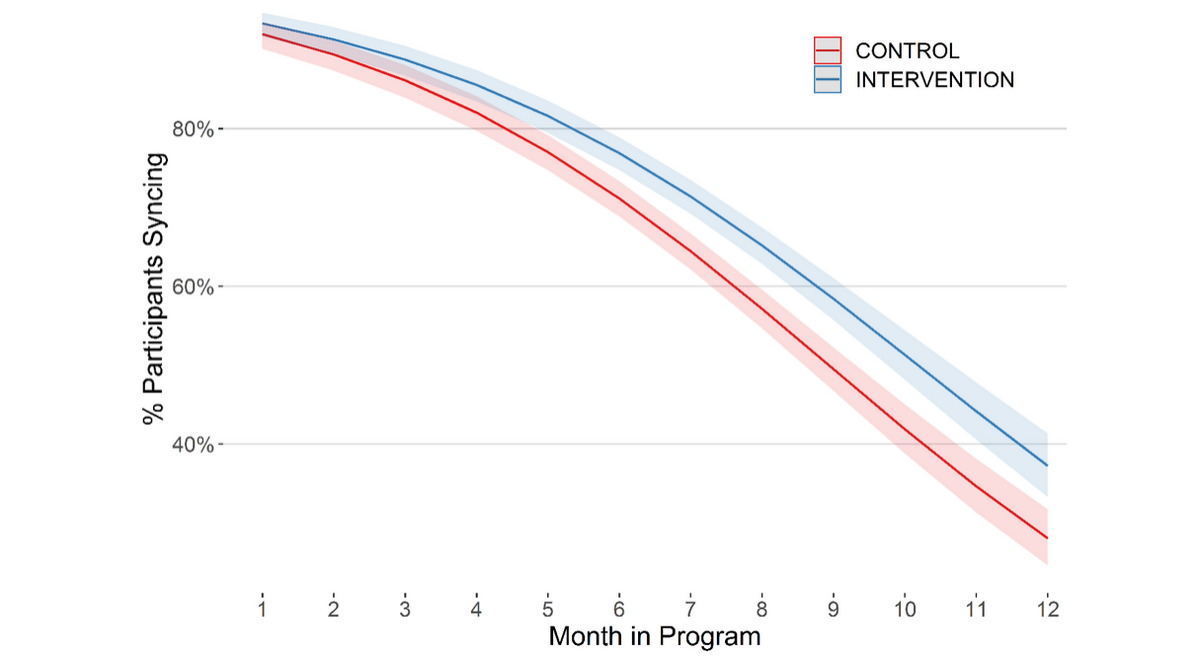
Percentage of participants who synced their Fitbit within the last 30 days by month and arm.

Of the 178 *Stay Strong+Coaching* arm participants, 70.8% (n=126) completed at least 2 coaching calls, and 56.7% (n=101) completed all 3 phone calls. However, participants in both groups increasingly failed to sync their Fitbit devices over their 12-month program (Figure 2). At 9 weeks, soon after coaching ended, there was no difference in syncing rates between the two groups (*P*=.14). By 6 months postbaseline, 60.3% (108/179) and 68.5% (122/178) of participants in *Stay Strong* and *Stay Strong+Coaching*, respectively, synced their Fitbit data. This difference was reflected by participants in *Stay Strong+Coaching* having higher odds of syncing their data compared to participants in *Stay Strong* (OR 1.36, 95% CI 1.17-1.58; *P*<.001). This difference was not sustained at 12 months postbaseline: rates were comparably low with 33.0% (59/179) and 38.2% (68/178) in *Stay Strong* and *Stay Strong+Coaching*, respectively, syncing their Fitbit data.

### Satisfaction With Stay Strong

The 12-month satisfaction survey was completed by 51.8% (185/357) of participants across the two programs, 89.7% (166/185) reported being “likely” or “extremely likely” to recommend their program to another veteran, and 69.7% (129/185) agreed or strongly agreed that their program was a benefit to their overall health. Overall, when asked if they did *not* like wearing their Fitbit, only 13.5% (25/185) strongly agreed or agreed. Though only 24.9% (46/185) of respondents strongly agreed or agreed that they found it difficult to sync their Fitbit using a desktop computer, 82.2% (152/185) strongly agreed or agreed that they would rather sync their Fitbit data using a smartphone than a desktop computer.

## Discussion

### Summary of Findings

This is the first completed large-scale trial of an mHealth intervention using wearable PA tracking devices (Fitbit) and a smartphone app among veterans. Adding automated and phone-based human coaching to the *Stay Strong* mHealth program (*Stay Strong+Coaching*) did not improve PA levels compared to baseline, nor compared to the *Stay Strong* program alone among US veterans of recent wars. Specifically, the rate of changes did not show difference between arms in Active Minutes, step counts, patient activation, and weight at 6 months or 12 months. In *Stay Strong+Coaching*, 70.8% (126/178) of participants completed at least 2 of 3 planned coaching calls in the first 9 weeks of the program but, like participants in *Stay Strong*, significantly decreased the frequency of syncing their Fitbit device to the point where over 60% (230/357) of trial participants had missing PA data 12 months postbaseline.

### Program Adherence and Missing Data

The completion of coaching calls was high: 70.8% (126/178) completed at least 2 calls, and over half (101/178, 56.7%) completed all 3 phone calls, even with the third call being optional. Other than the coaching calls for *Stay Strong+Coaching*, participants completed their program without human interaction. We attempted to reach participants who had not synced their Fitbit data within 7 days of the 12-month program ending; up to 9 phone calls were made over a 3-week period, with one follow-up letter. Sustaining long-term adherence to and engagement with mHealth interventions without human contact is challenging [26,57,58]. The rate of data syncing was relatively high at 3 months (317/357, 88.8%; Figure 2) in our trial, which is the time period evaluated in many published mHealth trials. However, the percentage of participants who synced their data by 12 months was low (127/357, 35.6%) for both arms. One potential explanation for this is that participants were asked to use a dongle plugged into a computer’s USB port with Fitbit Connect software to bypass direct syncing using Fitbit’s proprietary app installed on their smartphone. This process was not as efficient as using Fitbit’s app directly for syncing their device; in fact, the Connect software is no longer supported. Ethics oversight required use of the Connect software, however, to minimize the possibility of personal information (eg, name, locations, information from contacts lists stored on their smartphone) being accessed and stored by Fitbit. At 6 months, participants in *Stay Strong+Coaching* were more likely to sync their Fitbit data, suggesting that the added telephone-based health coaching with automated weekly personalized goal messages and personalized and standard motivational messaging may have helped retain participants for a longer period.

### Participant Characteristics and mHealth Interventions

*Stay Strong* was targeted specifically to OEF, OIF, and OND veterans who tend to be below-average age compared to the general veteran population. The average age of our participants was under 40 years old; this is much younger than the general veteran population, more than half of whom are over 60 [59]. Recruitment goals were met more quickly than initially planned, indicating a high level of initial enthusiasm for the study and potential ease of virtual enrollment procedures. However, despite high baseline PA levels, our participants reported lower quality of life (117/357, 32.8% reported fair or poor health) compared to the general US adult population (typically below 20%) [60,61]. Our study participants also had a high burden of mental health and other comorbidities; one-third to over half reported clinical depression, moderate or severe pain, or had a PTSD diagnosis. These conditions all present potential challenges to maintaining or increasing PA levels [4]. Other research has identified potential risks of developing mHealth interventions that are too complex, that may be inattentive to user needs and capabilities, and may leave vulnerable patients behind [62], perpetuating health disparities. Less healthy and poorer individuals may be least likely to use interventions using wearable devices [63].

### mHealth Interventions for Physical Activity

PA levels decreased by 41-65 active minutes at 12 months compared to baseline. However, PA declines among *Stay Strong+Coaching* participants largely occurred after the first 6 months, while *Stay Strong* participants continuously declined throughout their 12-month program. Thus, the additional lifestyle coaching support may have helped sustain PA levels longer compared to *Stay Strong* alone when any possible lasting effects subsided. Our findings are consistent with others who found that PA decreases over time in studies employing accelerometers as an intervention strategy [26]. We found no evidence of reactivity where participants may have been motivated to increase their PA levels during their baseline week even with a “Do Not Remove” sticker that covered their device so they would not see their data; on average, PA did not significantly decrease in the second week postbaseline.

This study marks a significant contribution to the mHealth literature. Our negative findings should be viewed in context of having an active comparator: both arms of the trial provided devices and a smartphone app to support PA. Further, this was a randomized trial of a relatively long-term program (12 months), drawing from a large national sample of OEF, OIF, and OND veterans who consented and enrolled online with no in-person assessments or interactions. *Stay Strong* was designed based on a fully described theoretical framework [36], which few apps do [64]. Furthermore, we followed participants for 12 months, which is longer than most other published trials [9,12,26,57,58]. Further development and testing are needed to continue to find interventions to help people increase and, importantly, sustain PA levels. Higher intensity and dose of human coaching may help. All human coaching was completed within the first 9 weeks; timing calls based on synced data (eg, when PA decreases or a participant fails to sync in a period of time) may help bolster levels when an individual is waning in their efforts or encountering new challenges, or increasing the number of calls over a longer period of time. Further, it is important to note that the content of the coaching calls may need to shift over time as participants lapse in and out of maintenance or receive new threats to their lifestyle modifications. Thus, behavioral strategies used to initiate behavior change, like increasing PA, are likely different from those needed to sustain gains over time. Our theoretical model was based on supports needed to initiate behavior change. We did not implement human coaching supports to maintain changes. A recent systematic review of behavior change techniques supports this hypothesis [65]. Although goal setting and self-monitoring of behaviors were important in both short- and long-term behavioral change, long-term behavior change also benefited from additional behavioral supports such as giving feedback on the outcome of the behavior, adding objects into the participant’s environment, receiving social support, and problem solving. These long-term techniques are likely hard to communicate or practice without providing human coaching over a longer time.

### Human Coaching to Strengthen mHealth Interventions

To our knowledge this is one of the first studies to assess the addition of coaching components on PA as an add-on component to an mHealth intervention with objective self-monitoring and feedback. The goals of our coaching strategy were to aid initial engagement and help keep interest in the mHealth intervention fresh and interesting for participants so they would continue to participate and, thus, enhance impact of the mHealth program [66]. Therefore, we frontloaded human coaching to occur within the first 9 weeks of the program. Much of the literature compares multimodal such as coaching + mHealth + objective self-monitoring to usual care or weak, inactive comparators such as an educational comparator [26,67]. Such designs make it impossible to tease out the independent contribution of coaching to mHealth engagement. Moreover, in much of the literature, mHealth plays a supportive role in the intervention with coaching as the central component. In *Stay Strong*, the mHealth platform is the central intervention component and human coaching is subordinate (ie, only 3 sessions in first 9 weeks of a 12-month mHealth program). Other studies have demonstrated that approaches that integrate coaching have more robust outcomes, and this was a central hypothesis in our study. The current literature is not adequate to address the independent contribution of coaching on mHealth interventions aimed at increasing PA [26,67].

### Role of Motivational Messages

Our barrier-specific messaging was based on a twice-administered survey (baseline and again 6 months later) of barriers such as lack of time, asking participants to choose up to four possible PA barriers they would encounter. This allows targeting messages to specific barriers. However, more recent advances with microtailoring based on season, geographic location, momentary mood, personal characteristics, employment and parenting demands, or other life circumstances would provide more actionable, meaningful, and potentially more motivating messages. Further tailoring to PA levels may also be effective, such as messaging when there is a gap in synced data [68], and the addition of more strategies to motivate and engage [69]. A challenge with mHealth interventions is that the novelty of the intervention may be motivating for a short period, but after the novelty wears off, the interventions lose their effectiveness. This is true for messaging as well; though we varied the time and day of our messages to help make them “fresh,” they were not timed based on any specific attributes or preferences of the participant. Another component to consider is the addition of an online community to increase engagement [70,71], though one systematic review only found trials that lasted 14 weeks or less [72], well before our participants, 64% (230/357) of whom were still syncing at 6 months, stopped syncing. Additionally, we did pilot approaches with veterans and used their feedback to guide development of the *Stay Strong* interventions. User-centered design approaches that more deeply involve potential participants in design through evolving rounds of development [73-75] to inform outcomes [76,77], information displays, and message content and timing may result in higher intervention durability and better outcomes.

### Study Limitations

This trial has several limitations. This trial was designed to assess outcomes between two mHealth interventions (*Stay Strong* vs *Stay Strong+Coaching*); this design precluded our ability to assess and, thus, compare change in PA among veterans without any mHealth intervention. However, nearly half of participants reported prior experience using a PA device, indicating its widespread use, which makes it challenging to require participants *not* to use a device while participating in a trial. Our primary outcome was Active Minutes, a proprietary measure captured by the Fitbit device that captures moderate or vigorous exercise levels in bouts of at least 10 minutes. This metric may have been confusing and, thus, demotivating for some participants who may not have fully understood why they were not getting “credit” for exercise if they failed to get their heart rate up for a long enough period. On the other hand, Fitbit also displayed step counts, which is a well-known and commonly used metric. Our findings are based on a minority of participants who synced data at 12 months postbaseline. Syncing frequency was our only indication of adherence to the *Stay Strong* app. Unfortunately, we did not have the ability to build in other measures of adherence or engagement at the participant level including, for example, time spent in the app. Multiple alternative models did not reveal any clear bias between participants who were lost to follow-up versus those who were included in our outcome analyses. Baseline PA levels were quite high among our study participants; over half met the minimum standard of 150 moderate or vigorous minutes of PA per week at baseline, which was surprising, given earlier indications of low PA levels among veterans [3]. Exploratory analyses did not support the possibility that participants may have increased their PA at baseline compared to a true “usual” level, even with a “Do Not Remove” sticker covering their Fitbit display.

### Conclusions

Although research has shown mHealth to have potential for promoting health behavior change, long-term participant adherence to study protocols and sustained engagement with mHealth interventions remains a challenge [24-26]. Our trial results have important implications for future research in this arena. Over 12 months, participant adherence to study protocols across both *Stay Strong* programs declined over time, as did PA levels. Although more *Stay Strong+Coaching* participants synced their Fitbit at 6 months compared to *Stay Strong* alone, we found no significant differences in PA between groups at 9 weeks, shortly after coaching ended for the *Stay Strong+Coaching* participants, nor at the end of the program (12 months). If we had less loss to follow-up at 12 months, we may have seen intervention effects. Continuing to develop ways to optimize content and type of automated and intensifying human health coaching informed by evidence-based behavior change techniques are strategies to explore to realize the full potential of mHealth.

## Abbreviations

ANCOVA: analysis of covariance
HIPAA: Health Insurance Portability Accountability Act
mHealth: mobile health
OEF: Operation Enduring Freedom
OIF: Operation Iraqi Freedom
OND: Operation New Dawn
PA: physical activity
PAM: Patient Activation Measure
PTSD: posttraumatic stress disorder
VA: Veterans Affairs
VHA: Veterans Health Administration
